# Client experiences of structural stigma in mental healthcare settings

**DOI:** 10.1371/journal.pmen.0000493

**Published:** 2025-12-05

**Authors:** Heather Stuart, Stephanie Knaak

**Affiliations:** 1 Department of Public Health Sciences, Queen’s University, Kingston, Ontario, Canada; 2 Mental Health Commission of Canada, Ottawa, Ontario, Canada; 3 Department of Community Health Sciences, University of Calgary, Calgary, Alberta, Canada; Florida State University College of Nursing, UNITED STATES OF AMERICA

## Abstract

Structural stigma in mental healthcare is reflected in organizational cultures of care that are experienced by service recipients as negative and disempowering. This includes pathways to care, clinical practices, shared ways of thinking, patterns of communications, and patterns of interaction. We report on (a) the prevalence of structural stigma experienced by a national sample of 818 clients 18 years of age or older receiving mental or substance use care in the past two years, and (b) the testing of two new scales to assess structural stigma in mental healthcare settings; The Coercive Care Scale and the Person-centred Scale. Structural stigma experiences were drawn from a prior focus group study. Survey respondents for psychometric testing were sampled from a large Canadian polling firm using a national sample. Exploratory and confirmatory factor analyses were performed on random split halves of the sample. Respondents reported a wide range of experiences indicative of structural stigma. Over half reported a punitive or coercive care experience and almost half reported care that was not person-centred. Factor analyses resulted in two sub-scales: one measuring 12 aspects of coercive care (The Coercive Care Scale, alpha = .92) and the other measuring 8 aspects of person-centred care (The Person-centred Scale, alpha = .86), both with excellent reliability. These scales are unique in that they are grounded in the personal experiences of service users and characterize an entire episode of care (rather than a specific provider/client interaction) from the perspective of the individual receiving care. Items can be used to target specific issues for quality improvement initiatives or aggregated into their sub-scales to monitor cultural change over time. Tools such as these can foster efforts to identify and reduce structural stigma in mental healthcare settings and assess the effectiveness of anti-stigma interventions designed to improve stigma cultures.

## Introduction

People with mental and substance use disorders frequently report experiencing stigma (negative or prejudicial attitudes or discriminating behaviours) in mental health care settings. Indeed, people who seek services from mental health professionals, often rate them as among the most stigmatizing of all groups [1]. Recurrent themes include feeling punished, patronized, humiliated, spoken to as if they were children, being excluded from treatment decisions, and being assumed to lack capacity to make treatment decisions. Other problems include prognostic negativism on the part of clinical staff (giving the impression that recovery is not possible); not being given sufficient information about their illness, treatments and side effects to make informed decisions; experiencing the unspoken threat of coercive care; and in some instances experiencing overtly coercive care such as involuntary hospitalization, seclusion, or forced treatment [1–3]. In an early qualitative study of people living with schizophrenia in Germany, for example, clients reported feeling stigmatized by their providers’ lack of interest in them as a person, the prognostic negativity with which their diagnoses were communicated, and a cavalier use of medications that had socially important side effects such as weight gain, that complicated their social interactions and reduced their self-esteem. They felt reduced to their illness-related deficits and were disheartened by the explicit and implicit messaging that they would be ill for the rest of their lives and may ultimately die of suicide [4].

In addition to negative client/treatment provider interactions such as those described above, researchers have become increasingly interested in the broader social and structural factors creating stigmatizing climates of care that shape professional interactions with service users [5]. Termed ‘structural stigma’, it has its roots in the concept of institutional racism, which recognizes the roles played by institutions, through policies and cultural ideologies in perpetuating disadvantage. Structural stigma has been defined as the “societal-level conditions, cultural norms, and institutional policies that constrain the opportunities, resources, and wellbeing of the stigmatized” [6] (p. 2). Hermaszewska et al. [7] have raised concerns about whether conventional anti-stigma efforts that ignore structural forces can authentically change the way in which people with mental illnesses are treated and how enduring that change will be. Rather than, or in addition to approaches that educate the public, they call for greater attention to structural elements of stigma; specifically, how it is interwoven into the fabric of social and political systems to exclude, exploit and control others.

Structural stigma in mental healthcare settings is reflected in stigmatizing cultures of care that are experienced as negative and disempowering by service users. Within mental healthcare settings, this includes pathways to care, clinical practices, shared ways of thinking, patterns of communications, and patterns of interaction [8]. Some of the broader structural forces that create stigma cultures in mental healthcare settings include policy decisions that underfund mental health services and mental health research, professional educational curricula that underrepresent mental health teaching, hidden curricula that dissuade medical students from entering psychiatry, mental health laws that support coercive care practices, unwelcoming and scary physical spaces, and clinical care practices that fail to promote recovery oriented care [9,10]. Recovery oriented care encourages individuality; promotes positive portrayals of people with a mental illness; fights stereotypes, prejudice, and discrimination; focuses on strengths rather than deficits; uses hopeful and non-stigmatizing language; offers a variety of treatment options encouraging client choice; and supports risk-taking even when failure is a possibility. In a recovery-oriented environment, clients and significant others are actively involved in decision making and considerable emphasis is placed on helping clients develop valued and meaningful social roles [11]. Trauma-informed care has many of the same processes but reflects a broader conceptualization of the role of traumatic events stemming from social and political contexts. In healthcare environments trauma-informed approaches recognize the potential for healthcare systems to create powerlessness, lack of agency, and punitive or coercive care practices. Trauma-informed approaches require recovery-oriented environments for all involved in the care process—clients, staff, family, friends, and other allies [12].

Both recovery oriented and trauma-informed practices require environments that are non-stigmatizing and person-centred. In a Canadian focus group study of structural stigma experienced in healthcare settings conducted under the auspices of the Mental Health Commission of Canada, participants reported a range of negative and disempowering experiences indicative of stigmatizing cultures [13]. For example, mental healthcare staff were not seen as relating to clients as people, but to their diagnostic labels, with little awareness of recovery principles. Participants described a power differential between staff and those receiving care, and an environment that was overly controlling and, at times, punitive or coercive. They experienced a lack of transparency about their treatments and their side effects, a lack of communication about the processes and outcomes of care, and poorly designed physical spaces. Heavy use of security personnel in places such as emergency rooms, was experienced as frightening and trauma inducing. Diagnostic overshadowing was described where a person received inadequate or delayed treatment for a physical condition because it was misattributed to the underlying mental condition. People who experienced a substance use disorder, even those in recovery and on methadone maintenance, had a particularly difficult time. As several participants wore two hats—lay and professional—they also provided perspective on the struggles experienced by the wounded healer. They noted the culture of healthcare was such that providers must be resilient, tough, and not experience mental or substance use problems. It was an unspoken rule that health providers could not have mental health issues, or if they did, certainly could not speak about them.

An important first step in reducing stigma cultures is to assess the scope and magnitude of stigma experienced by people with a mental illness or substance use disorder who are mental healthcare recipients. Two measurement approaches have been used [5]. First, policies (typically country, state, or municipal policies) have been content analyzed to identify the presence of structural stigma components, but this has not been done in the context of healthcare systems. While policy analysis represents an objective measure, content analyses of formal policies miss the unwritten customs and procedures that undergird more informal institutional practices such as those indicative of stigma cultures in healthcare settings. Secondly, attitude surveys have been used to assess the public’s attitudes toward members of stigmatized groups.

Modgill and colleagues provide an example of how attitude surveys have been adapted for use in healthcare settings [14]. Working under the auspices of the Mental Health Commission of Canada, they developed and psychometrically tested a three-factor scale to assess health provider attitudes and behavioural intentions toward people with a mental illness. The first factor addressed the attitudes of healthcare providers and included items such as “More than half of the people with mental illness don’t try hard enough to get better”. The second factor measured disclosure and help-seeking including items such as “I would be reluctant to seek help if I had a mental illness”. The third factor examined social distance including items such as “If a colleague with whom I work told me they had a managed mental illness, I would be as willing to work with him/her”. The authors recognized that a limitation of this approach was the possibility that social desirability response sets would bias results yielding underestimates. A second limitation is that reported attitudes may not capture behavioural responses—a problem that can only be rectified if clients are asked about their experiences directly.

The growing interest in structural stigma has generated a need for measurement instruments. The bulk of tools designed to measure aspects of organizational cultures both inside and outside of healthcare settings have been directed to the self-reports of employees (rather than clients) and focussed on organizational structures and practices, such as leadership, communications, or quality of work life. Reviews of existing instruments have found them to be limited in scope, ease of administration, and scientific properties. For example, Jung and colleagues [15] reviewed instruments measuring organizational cultures (not specifically healthcare cultures). Of the 70 instruments identified, none had robust reliability or validity data, and none took the perspective of the client.

To gain a better understanding of client experiences with structural stigma and to identify constructs that could be used to measure structural stigma in healthcare settings, researchers working with the Mental Health Commission of Canada conducted a focus group study of mental health service users [16]. An important theme that emerged from this analysis was that the culture of care was “broken”. Care routines were experienced as demeaning, dehumanizing, robotic, and prison-like—not person centred, recovery oriented, or empowering. Participants were also asked to describe the characteristics of an ideal measure that could be used to monitor structural stigma in healthcare settings. Characteristics included being grounded in the experiences and priorities of service users; client-directed such that service-users complete the measure rather than health professionals; holistic capturing the service user’s overall care experience rather than a specific care process or provider/client interaction; person-centred to address the extent to which the care environment meets client’s needs, is empowering, affirming, and recovery-oriented; generalizable to a broad range of health and mental health settings to ensure that physical, social, and mental health needs are met in supportive environments across the full spectrum of healthcare settings; and psychometrically sound. Qualitative data from this focus group study were used to develop two measures—one to assess structural stigma in general healthcare settings, and one to assess structural stigma in mental healthcare settings. An earlier paper reports on the factor structure and reliability of the 23-item *Stigma Cultures in Healthcare Scale* [16] In the current paper we report on (a) the prevalence of aspects of structural stigma experienced by a national sample of clients receiving mental health care, and (b) the potential of these items to yield a psychometrically sound measurement scale that will fill an important gap in the measurement literature

## Methods

### Ethics statement

Ethics clearance was provided by the Queen’s University Health Sciences and Affiliated Teaching Hospitals Research Ethics Board on November 29, 2021 (Reference number 6035081). Deidentified anonymous survey data collected by an external survey firm were given to the authors for this secondary analysis. Informed consent procedures were conducted by the external survey firm. For example, all panelists had previously provided informed consent to receive survey invitations and consented by completing the survey.

### Study design

Details of item generation and survey data collection have been described elsewhere [16]. Briefly, items were generated based on both inductive (focus group) and deductive (literature review) processes. Initially, 20 focus group participants with lived experience of a mental or substance use disorder described personal experiences of structural stigma in healthcare settings. Focus group data were qualitatively analysed to reveal specific constructs. A literature review was then undertaken to map items from existing questionnaires and scales onto these constructs. Additional items were developed where existing items were inadequate or non-existent. Items were given back to focus group participants for content validation and readability statistics were used to ensure that the highest reading level of any item was not above grade 8 (the majority were grade 6 or below). Items were rated on a 4-point agreement scale with no neutral option. Additional options allowed for not appliable, don’t know, and prefer not to say.

The Mental Health Commission of Canada commissioned an external polling firm to conduct a national survey from their online polling panel. Data were collected in February 2022. To be included in this analysis, respondents had to be living in Canada, 18 years of age or older, report a mental or substance related condition, and have received mental health care in the prior two years (n = 818). To build the sample, respondents were randomly selected from among the online panelists who met the inclusion criteria. Eligible survey respondents were then quota sampled proportionate to the 2016 Canadian census profile by region, age, and gender. Once a quota was reached, additional completed surveys were excluded from the dataset for that group. This provided a heterogenous sample that was broadly reflective of Canada’s regional and demographic makeup [16].

Respondents were asked to specify a mental health care experience from the past two years then answered questions about stigma they may have experienced when receiving that care. Several items were reverse coded to avoid an overly negative frame. To meet the first objective, prevalence was defined as the proportion of the sample reporting a stigma experience, measured as those who agreed or strongly agreed divided by the total n-size. Because non-response options and missing values were not included in the numerator, but were included in the denominator, prevalence estimates will be under-estimated. Estimates were weighted using the weight variable provided by the polling firm to provide a more representative sample. To meet the second objective, the sample was randomly split. Exploratory factor analysis was conducted on half of the sample and confirmatory factor analysis on the other (n = 409 in each split half). For the exploratory factor analysis, principal axis factoring using a polychoric correlation matrix for ordinal data and robust standard errors was used. Confirmatory factor analysis used structural equation modelling with maximum likelihood and robust errors. Reporting of results followed the approach outlined in Stata’s Structural Equation Modelling Reference Manual Release 17 [17].

## Results

Table 1 shows the socio-demographic characteristics for respondents who had received care in the previous two years for a mental health or substance use issue. Most were seen by family doctors (76%), in outpatient clinics (30%), or in walk-in clinics (26%). By design, most respondents came from the most populous provinces, Ontario and Quebec. Almost half (45%) were between the ages of 18 and 35 and more than half identified as female. Almost a third of the sample had a bachelor’s degree and another 25% had a postsecondary diploma. The majority (62%) were employed or self-employed. Seven percent were unemployed or laid off and 6% were on disability. With respect to the timing of their diagnosis, almost half had been diagnosed more than two years prior to the survey.

**Table 1:** Sample Characteristics (n = 818).

Item	Weighted %*
Province of residence	
British Columbia	12.4%
Alberta	12.1%
Prairies	7.5%
Ontario	37.9%
Quebec	22.6%
Atlantic Canada	7.5%
Age group	
Between 18 and 24	18.1%
Between 25 and 34	26.4%
Between 35 and 44	19.1%
Between 45 and 54	17.3%
Between 55 and 64	11.8%
Between 65 and 74	6.1%
74 or older	1.2%
Gender	
Male	42.5%
Female	56.5%
Non-binary	1.0%
Highest level of education	
Less than high school	2.4%
High school	15.2%
Some Postsecondary	11.6%
Postsecondary diploma	24.9%
Bachelor’s degree	31.7%
Master’s/professional	11.8%
Doctorate	1.0%
Other	1.2%
Prefer not to say	0.2%
Current employment status	
Employed/self-employed	62.0%
Unemployed/laid off	7.0%
Student	11.5%
Retired	10.2%
Disability	5.8%
Other	3.2%
Prefer not to say	0.3%
Length of time since most recent diagnosis	
Less than a year	23.1%
1–2 years	27.0%
More than 2 years	48.8%
Don’t know	1.0%
Prefer not to say	0.1%
Mental Health care experiences last 2 years (multiple response)	
Hospitalized mental health	12.2%
Hospitalized substance use	6.1%
Emergency visit mental health	15.2%
Emergency visit substance use	5.7%
Family doctor mental health	75.8%
Family doctor substance use	9.7%
Outpatient clinic/diagnosis mental health	30.4%
Outpatient clinic/diagnosis substance use	7.2%
Walk-in clinic mental health	25.5%
Walk-in clinic substance use	6.8%

* N-sizes are not shown as these will not correspond to the weighted percents.

### Objective 1: Estimating the Prevalence of Perceived or Experienced Stigma

Table 2 presents the prevalence of structural stigma experienced across the 23 survey items that were developed from the qualitative study described above. As some of the items were reverse coded, the prevalence reflects the proportion of respondents reporting that they perceived or experienced structural stigma with reference to each care component. Patient advocacy issues were the most frequently reported with almost half of the sample indicating that they were not told about patient advocacy services or how to complain about their treatments. The next most frequently endorsed questions pertained to decision-making (e.g., questions were invasive, not encouraged to asked questions, not being told about side-effects, etc.). The next most frequent items pertained to the nature of the provider client relationship, (e.g., feeling powerless, ashamed, devalued, no hope for recovery etc.). Finally, several items pertained to the nature of the care provided as being experienced as punitive or coercive (e.g., pressure to take medication, threatened to be admitted against will, too much security, etc.). The count of reported stigma experiences reveals that most clients (83%) could recount at least one stigma experience and almost half (44.2%) could identify 4 or more. The median was 2.5 indicating that half of the sample could recount experiencing approximately 3 or more stigma experiences when receiving mental health care.

**Table 2:** Prevalence of perceived or experienced structural stigma in mental healthcare settings (n=818).

Items	Perceived or Experienced Stigma (weighted)
Not told about Patient Advocacy Services	47.5
Not told how to complain about my treatment	45.6
Felt powerless	31.4
Some questions were invasive	26.4
Not encouraged to ask questions	21.5
Not told about medication side effects	20.5
Made to feel ashamed of my condition	20.0
My rights were not explained	19.4
Felt devalued	19.3
Pressure to take medication I did not want	19.2
The space was not comforting	18.4
My rights were not respected	18.0
Made to feel there was no hope for recovery	16.7
Did not get the care needed	15.9
Made to feel unreliable	15.8
Not well taken care of	15.5
Made to feel untrustworthy	15.1
There was too much security	15.0
Security staff were scary	14.6
Views not respected	14.3
Heard demeaning language	12.8
Not treated with respect	12.7
Threatened to be admitted against my will	12.7
Count of Stigma Experiences (Median = 2.5)	
0	17.0
1	13.8
2	14.9
3	10.1
4-23	44.2

### Objective 2: Factor structure and reliability

As recommended by Morgado et al., both an exploratory and confirmatory factor analysis was undertaken as our split sample sizes exceeded the recommended 10–1 ratio of subjects to survey items [18]. For all analyses, items were coded such that higher scores reflected higher levels of reported structural stigma and “no agreement” was coded as 0. Regarding the exploratory factor analysis, Bartlett’s test of sphericity and the Kaiser-Meyer-Olkin measure of sampling adequacy indicated that the data were sufficiently correlated to be suitable for factor analysis (KMO.928, Bartlett 4978.521, df = 253, p < 001). A polychoric correlation matrix suitable for ordinal data was used with principal axis factoring and oblique rotation. Three eigenvalues exceeded the recommended threshold of 1.0 (9.5, 2.3, and 1.0) with a cumulative variance explained of 93% (69%, 16%, and .1% respectively). Rotated items loaded onto one of three factors. Parallel analysis also yielded three factors with adjusted eigenvalues of 8.3, 2.3, and 1.1. Examining the content of the items, Factor 1 deals with aspects of coercive care and its effects on the individual. Factor 2 deals with aspects of person-centred care and Factor 3 deals with advocacy rights. The first two factors showed excellent reliability with alpha’s exceeding .80 (which is preferred for a development sample). The reliability of the third factor was weak for a development sample. Given the low eigenvalue, the small amount of cumulative variance explained, and the less-than-ideal reliability, Factor 3 was dropped from further analysis. Table 3 summarizes the factor loadings and alphas for the three factors.

**Table 3:** Factor loadings exploratory factor analysis (n = 409).

Exact Item Wording	Factor 1 Coercive Care	Factor 2 Person-Centred Care	Factor 3 Advocacy Rights
I was made to feel ashamed of my condition	.65		
I felt some questions were invasive	.62		
I was threatened to be admitted against my will	.72		
I felt pressure to take medication I did not want	.64		
I felt powerless	.56		
I heard demeaning language	.84		
I felt devalued	.77		
I was made to feel untrustworthy	.81		
I was made to feel unreliable	.82		
There was too much security	.71		
Security staff were scary	.76		
My rights were not respected	.60		
Cronbach’s alpha	**.92**		
I got the care I needed		.74	
I was well taken care of		.71	
I was treated with respect		.75	
I was made to feel there was hope for recovery		.47	
I was encouraged to ask questions		.59	
My views were respected		.68	
I was told about medication side effects		.49	
The space was comforting		.60	
Cronbach’s alpha		**.86**	
My rights were explained			.50
I was told how to complain about my treatment			.81
I was told about Patient Advocacy Services			.79
Cronbach’s alpha			**.73**

### Confirmatory factor analysis

Based on the exploratory results, we tested a 2-factor model initially allowing for correlations between factors. Subsequent modification indices were used to identify potentially missing paths reflecting correlated error terms. These were allowed only when items were conceptually similar, if modification indices were large (over 10), and if the goodness of fit statistics were substantially improved by adding the correlated error terms. Ultimately, we allowed for correlated error terms between the two items addressing security (Security staff were scary and there was too much security), based on a modification index of 74.4. The model was refit and modification indices were again reviewed. Though there were several additional paths that could have been added (with modification indices in the range of 16–25), these did not substantially improve the fit of the model.

Table 4 summarizes the standardized factor loadings and the correlation between each item and the factor (shown as R^2^) in the final model. The R^2^ for the overall model fit and the Standardized Root Mean Squared Residual (SRMSR) are also shown to assess goodness of fit. In this model, the standardized factor loadings were strong, ranging from .47 to .86 and there was a moderate correlation between the factors (.53). (Note that the correlation is positive as the person-centred care items were reverse coded.) The R^2^ showed that the model accounted for over 99% of the variance in the factors, indicating an excellent fit, and the SRMSR of.06, was below the conventional threshold of 0.08 also indicating a good fit. Because robust errors were used, other conventional goodness of fit statistics, such as the RMSE, were not available. Raykov’s reliability coefficient showed that both factors had excellent reliability (.90 and .85 respectively).

**Table 4:** Standardized Factor Loadings, R^2^ values and Goodness of Fit Statistics for the 2-Factor Confirmatory Model.

Exact Item Wording	Factor 1 Coercive Care	Factor 2 Person-Centred Care	R^2^
I was made to feel ashamed of my condition	.71		.51
I felt some questions were invasive	.57		.32
I was threatened to be admitted against my will	.65		.43
I felt pressure to take medication I did not want	.58		.34
I felt powerless	.56		.31
I heard demeaning language	.81		.66
I felt devalued	.86		.74
I was made to feel untrustworthy	.86		.73
I was made to feel unreliable	.85		.22
There was too much security	.47		.26
Security staff were scary	.51		.45
My rights were not respected	.67		.51
I got the care I needed		.72	.66
I was well taken care of		.81	.64
I was treated with respect		.80	.64
I was made to feel there was hope for recovery		.58	.33
I was encouraged to ask questions		.66	.43
My views were respected		.66	.44
I was told about medication side effects		.50	.25
The space was comforting		.51	.27
Goodness of Fit Statistics			
R^2^ fit for the overall model			0.99
Standardized Root Mean Square Residual			0.61
Raykov’s Factor Reliability Coefficient	.90	.85	

**Fig 1:**
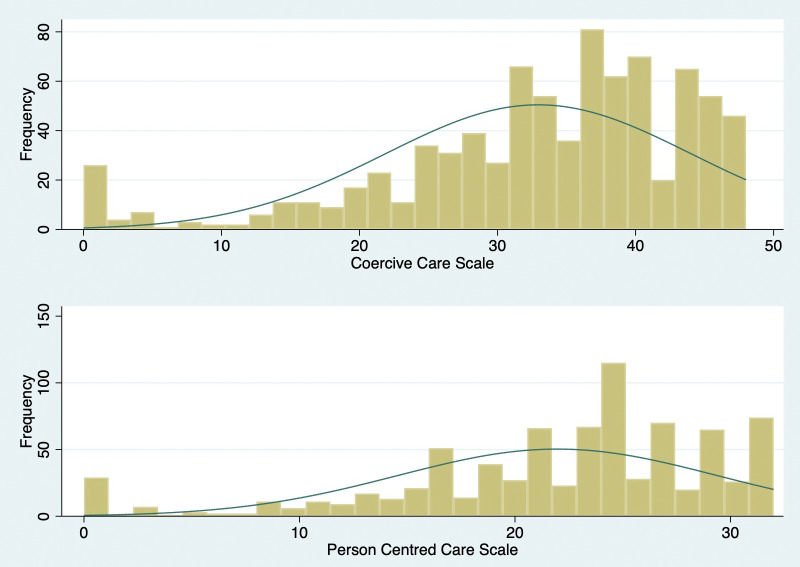
Distributions of Coercive Care and Person-Centred Scale Scores (n = 409).

We also tested the Coercive Care Scale, and the Person-Centred Scale for skewness and kurtosis. In both cases a chi-square of <.001 indicated that the distribution was a significant departure from normality (see Fig. 1).

Table 5 summarizes the number of structural stigma experiences reported in the full sample (n = 818) for each factor. More than half (56%) reported mental or substance use care that was experienced as coercive. In almost a quarter of the sample (22%), more than three coercive structural stigma experiences were reported. Almost half of the sample (46%) reported that their health care experiences were not person-centred, with almost one in five (19%) reporting more than two stigma experiences in this domain.

**Table 5:** Count of Structural Stigma Experiences Among Those Receiving Healthcare for a Mental or Substance Use Disorder in the Previous Two Years (n = 818).

Number of Stigma Experiences	Percent % (n)
Coercive Care	
0	44% (362)
1-3	34% (280)
4-6	9% (73)
7-9	7% (55)
10-12	6% (48)
Any	56% (456)
Mean = 2.1, SD = 3.2, Median = 0	
Person Centred Care	
0	54% (439)
1-2	27% (220)
3-4	9% (75)
5-6	6% (52)
7-8	4% (21)
Any	46% (379)
Mean = 1.3, SD = 2.0, Median = 0	

## Discussion

In this paper we have conceptualized stigma experienced in healthcare settings as a reflection of broad structural factors that can work synergistically to create organizational cultures that are experienced by service users as stigmatizing [16]. Over the past twenty years, there has been a growing understanding that negative (in this case stigmatizing) healthcare cultures result in poor quality of care and negative health outcomes. Attempts to shape cultures of healthcare are increasingly viewed as an important means of improving safety and performance outcomes and promoting high-quality person-centred and recovery-oriented care. As transforming cultures of healthcare has become viewed as a key aspect of health system reform, attempts to promote more caring cultures have grown [19]. Indeed, mental health system reformers and advocates have repeatedly called for massive transformations in the way healthcare is provided to people with mental health and substance use disorders using person-centred and recovery-oriented principles that, among other things, strive to reduce or eliminate stigma [11]. In recent years, this call has expanded to also acknowledge the importance of building trauma informed systems of care to address stigma [12].

It has been difficult to know how pervasive stigma is in the Canadian (or any other) healthcare system as there have been no coordinated attempts to systematically document its scope and magnitude. This research has provided new data demonstrating that clients report a wide range of stigma experiences when receiving mental health care ranging from lack of advocacy information, to negative interactions with care providers, and treatment approaches that are not person-centred, recovery-oriented, or are experienced as punitive or coercive. Over half of the respondents (56%) reported a care experience that was punitive or coercive and close to half (46%) reported the care they received was not person-centred. Considerable research has now documented that negative treatment experiences such as these can deter people with a mental illness or substance use disorder from seeking help. They can also damage interactions and therapeutic relationships with healthcare providers, diminish people’s motivation to complete treatment, and lead them to conceal issues to avoid stigma and improve their prospects of receiving better quality services [20].

Stigma cultures in mental health care systems need well developed and psychometrically tested instruments both to target specific areas for remediation and to use as an audit tool to monitor cultural change over time. The exploratory and confirmatory factor analyses revealed two strong factors—a 12-item factor pertaining to aspects of coercive care (which we named the *Coercive Care Scale*), and one 8-item factor pertaining to aspects of Person-Centred Care (named the *Person-Centred Care Scale*). Both scales had strong reliability coefficients. A third 3-item factor pertaining to advocacy rights was identified in the exploratory analyses, but the eigenvalue was close to the threshold of 1.0 and the reliability coefficient was 0.73, much lower than would be considered ideal for an exploratory analysis, likely due to the small number of factors pertaining to advocacy. Building out this scale to provide a more comprehensive assessment of advocacy related issues should be a priority for future research, as this was a key area of stigma experienced by our sample (47.5% indicated they were not told about Patient Services and 45.6% indicated they were not told how to complain about their treatment).

The confirmatory analysis using the items pertaining to the 2-factor solution showed a good fit, with 99% of the variance explained. Reliability coefficients for the two scales were excellent, at 0.90 and 0.85, respectively. The variance explained by each scale item ranged from 22% to 74%, suggesting that there may be room to reduce the scale sizes by eliminating the items with the least explanatory power, though this would require a fresh sample so must await further research. The scales are currently short (taking only 8 minutes to complete) and provide a broad overview of the nature of structural stigma in settings where people receive mental health care. Both scales departed from a normal distribution so should be used as continuous variables only with statistical procedures that are robust to violations of normality, or they should be recoded into categories. For example, we transformed the scale into a count variable indicating the frequency with which respondents reported having experienced structural stigma. Fifty-six percent of the sample reported that they experienced structural stigma in the form of coercive care practices and almost half 46% reported that they did not receive person-centred care. Now that these scales have been developed and psychometrically tested using factor analytic procedures, future research should prioritize testing measurement invariance and capturing validity evidence.

A limitation in our approach to estimating prevalence is that we did not have a randomly selected sample of Canadians with a mental or substance use disorder. Although the quota sampling and weighting were intended to minimize selection bias, panelists are unlikely to be fully representative of Canadians with mental or substance use conditions, particularly those with severe disorders. Secondly, given our inclusion of a “prefer not to say” option (and our inclusion of these in the denominator for prevalence calculations), it is likely that our prevalences are underestimates. However, selection bias should not diminish the usefulness of these tools for monitoring purposes as the same bias would be present each time. It is sobering to consider that the estimate that more than half of the respondents experienced coercive care may be an underestimate and underscores the need to mount initiatives to address structural stigma experienced in healthcare encounters.

Another consideration is our use of a 4-point agreement scale with no neutral option, rather than a longer (5 or 7 point) scale. While the shorter scale ensured that an unequivocal opinion was provided, fewer categories can reduce reliability [21], however, this may only be a problem for small heterogeneous samples. Moreover, there is prior research from randomized controlled trials showing that construct validity and discriminant validity are comparable across four- and five- point scales [22].

To date, most measurement tools evaluating components of healthcare delivery have been developed by researchers for researchers, with little input from people with lived experience of a mental or substance use disorder. Measures that focus on client perceptions of the care they have experienced are few, and when available, typically focus on a single provider-patient relationship (such as a doctor-patient relationship), a single location in the health system (such as a family practice clinic), or with a specific clientele in mind (such as cancer patients or elderly people in care homes). Evidence supporting validity and reliability is largely lacking [23]. To our knowledge, these are the first psychometrically tested scales that measure aspects of structural stigma in settings providing mental health care that are grounded in the personal experiences of service users and assess the entire healthcare experience. Because they are novel, we do not have an ability to situate our work in a broader scientific context, nationally or internationally. This will have to await further research.

Tools such as these can help foster efforts to reduce structural stigma in mental healthcare settings. Specifically, we envision them being used to monitor the effectiveness of organizational interventions such as staff workshops designed to improve trauma or person-centred care; reductions in the use of coercive practices such as seclusion, restraint, forced treatment, or use of security; and improvements to the physical spaces in which the treatments are provided. Scales could also be used as part of routine care reviews (e.g., at discharge), as a formal component of accreditation standards.

## Supporting information

S1 Data Data for Tables 1, 2, and 5 (n = 818). (XLSX)

S2 Data Exploratory Factor Analysis data (n = 409). (XLS)

S3 Data Confirmatory factor analysis data (n = 409). (XLS)
